# Leçons De Gynecologie Opératoire

**Published:** 1890-09

**Authors:** 


LEÇONS DE GYNÉCOLOGIÉ OPÉRATOIRE par VULLIET, Proféssseur à la Faculté de Médicine de Genevé, etc., et LUTAUD, Professeur libre de Gynécologie à l'ecole practique, etc. Deuxiéme édition entirément refondue, avec 200 figures intercalées dans le texte. 8vo, paper; pp. 500. Paris: A. Maloine, 91 Boulevard Saint-Germain.

The first edition of this work was reviewed at some length in this *Journal* for March, 1889, page 491. In less than a year's interval the second edition is now before the medical world, entirely revised and in part rewritten. Especially in operative gynecology have many additions been made,—as the procedures of Terrier and Leopold for the fixation of the uterus, Lawson Tait's perineorraphy, etc. The authors do not belong to the modern school of electro-gynecologists, but believe it impossible for an electric current to "electrolyse" all undesirable tissues, leaving the desirable tissues of the same histological structure unscathed and untarnished, save by the caustic action of the two poles. They do use, however, the interrupted current to bring about contractions of the uterus, thereby rendering sub-mucous fibromas sessile or pedunculated and, hence, easily removable.

Paper and print are all that can be desired, but the wood-cuts require full explanations to make them intelligible. W. C. K.

